# Nkd1 Functions as a Passive Antagonist of Wnt Signaling

**DOI:** 10.1371/journal.pone.0074666

**Published:** 2013-08-29

**Authors:** Diane Angonin, Terence J. Van Raay

**Affiliations:** Department of Molecular and Cellular Biology, University of Guelph, Guelph, Ontario, Canada; Oxford Brookes University, United Kingdom

## Abstract

Wnt signaling is involved in many aspects of development and in the homeostasis of stem cells. Its importance is underscored by the fact that misregulation of Wnt signaling has been implicated in numerous diseases, especially colorectal cancer. However, how Wnt signaling regulates itself is not well understood. There are several Wnt negative feedback regulators, which are active antagonists of Wnt signaling, but one feedback regulator, Nkd1, has reduced activity compared to other antagonists, yet is still a negative feedback regulator. Here we describe our efforts to understand the role of Nkd1 using Wnt signaling compromised zebrafish mutant lines. In several of these lines, Nkd1 function was not any more active than it was in wild type embryos. However, we found that Nkd1’s ability to antagonize canonical Wnt/β-catenin signaling was enhanced in the Wnt/Planar Cell Polarity mutants *silberblick* (*slb/wnt11*) and *trilobite* (*tri/vangl2*). While *slb* and *tri* mutants do not display alterations in canonical Wnt signaling, we found that they are hypersensitive to it. Overexpression of the canonical Wnt/β-catenin ligand Wnt8a in *slb* or *tri* mutants resulted in dorsalized embryos, with *tri* mutants being much more sensitive to Wnt8a than *slb* mutants. Furthermore, the hyperdorsalization caused by Wnt8a in *tri* could be rescued by Nkd1. These results suggest that Nkd1 functions as a passive antagonist of Wnt signaling, functioning only when homeostatic levels of Wnt signaling have been breached or when Wnt signaling becomes destabilized.

## Introduction

Patterning of the vertebrate embryo involves the coordinated efforts of multiple signaling pathways. The regulation of these pathways must be tightly controlled in order for normal development to proceed. Regulation can occur at several different levels, but one important mechanism is the ability of a signaling pathway to invoke a negative feedback loop. The Wnt signaling pathway is a major player in development and in homeostasis of stem cells [1–4] and several negative feedback regulators have been identified including Dickopff (Dkk), Wingful/Notum, Naked (1/2), Nemo, Axin2 and β-TCRP [5–14]. For the most part, these antagonists are very efficient at blocking both ectopic and endogenous canonical Wnt/β-catenin signaling, with the exception of Nkd1. We and others have previously reported that Nkd1 is an obligate target of Wnt signaling during vertebrate development and can inhibit both canonical and non-canonical Wnt signaling [12,14–16]. Subsequently, we found that Nkd1 inhibits canonical Wnt signaling by preventing the nuclear accumulation of β-catenin [13]. As β-catenin is restricted to the canonical Wnt pathway, it is unclear how Nkd1 antagonizes non-canonical or Wnt/PCP signaling, but likely involves Dvl [17]. In our investigations, we found Nkd1 to be very efficient at reducing ectopic Wnt signaling. For example, in the *bozozok* mutant, there is excess Wnt signaling from the ventro-lateral mesendoderm that reduces the size of the dorsal domain, which is manifested by a reduced or absent notochord [18–20]. Overexpression of Nkd1 rescues this phenotype, along with the eyeless phenotype induced by excess Wnt8a [12]. However, in the absence of excess Wnt signaling Nkd1 activity is less obvious.

The passive effect of Nkd1 may be a universal phenomenon. In flies, absence of Nkd results in a naked cuticle phenotype (hence its name) at larval stages, but even though *nkd* is expressed at multiple other stages of development in domains of active Wnt signaling, its loss of function does not appreciably affect these other Wnt signaling events [21]. Double knockout of *nkd1* and *nkd2* in mouse results in subtle alterations in cranial bone morphology, but are otherwise normal and fertile [22] and ubiquitous overexpression of Nkd1 in the mouse or in the fly embryo does not have any obvious consequences [15,17,21]. These results are consistent with our analysis of Nkd1 function in zebrafish. While careful examination reveals a role for Nkd1 in restricting Wnt-mediated D–V patterning, there is no obvious consequence to overexpression of Nkd1. However, in contrast to wild type embryos, severe loss-of-Wg signaling phenotypes can be induced in the embryo when Nkd is overproduced in Wg-compromised genetic backgrounds [17,21]. This suggests that Nkd activity is dependent on Wnt signaling itself and the use of compromised Wnt signaling mutants may be one avenue to further understand Nkd1 function.

In vertebrates it has been well established that the Wnt/PCP pathway can antagonize canonical Wnt/β-catenin signaling, but at what level in the pathway remains controversial [23–27]. Nonetheless, Wnt/PCP mutants may provide the necessary sensitivity to understand Nkd1 function. Along these lines, we evaluate how Nkd1 functions in several zebrafish Wnt mutant lines (canonical and non-canonical) and find that Nkd1 has the greatest activity in *silberblick* (*slb*/*wnt11*) and *trilobite* (*tri/vangl2*) mutants, both of which are specific for the Wnt/Planar Cell Polarity pathway. These mutant lines are sensitive to canonical Wnt signaling, but Nkd1 can rescue this sensitivity.

## Results

**Figure 1:**
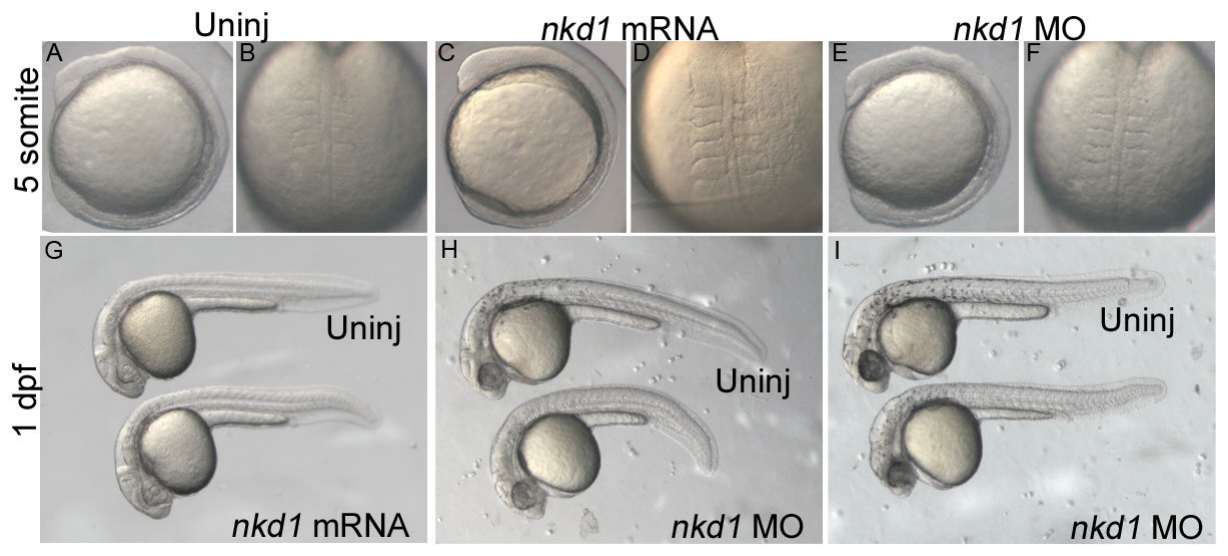
Nkd1 does not influence normal development and patterning of the early embryo. Injection of *nkd1* mRNA or morpholino (MO) does not have an obvious affect during early somitogenesis (A–F) or at 1 dpf (G–I). The number and width of somites in injected individuals is indistinguishable from uninjected embryos. At 1 dpf, injection of *nkd1* MO results in neural necrosis, which ranges from moderate (H) to more severe (I).

We have previously determined that Nkd1 is both necessary and sufficient to antagonize both canonical and non-canonical Wnt signaling [12]. However, in comparison to other Wnt antagonists such as Axin2 and Dkk1, the effect of Nkd1 is subtle [28–30]. As shown in Figure 1, overexpression or knockdown of Nkd1 does not have an overt phenotype during somitogenesis (Figure 1A–F) or at 1 day post fertilization (dpf) (Figure 1G–I). If knockdown of Nkd1 resulted in dorsalization of the embryo, we would have observed a characteristic elongated and ovoid morphology during early somitogenesis, characteristic of increased canonical Wnt signaling [31,32]. Unfortunately, morpholinos targeting *nkd1*, either at the start ATG or at a splice site, are toxic beginning at late somitogenesis, which is obvious by the neural necrosis at 1 dpf (Figure 1I). Despite this, there do not appear to be any significant differences between Nkd1 overexpression, Nkd1 knockdown and uninjected embryos at 1 dpf.

**Figure 2:**
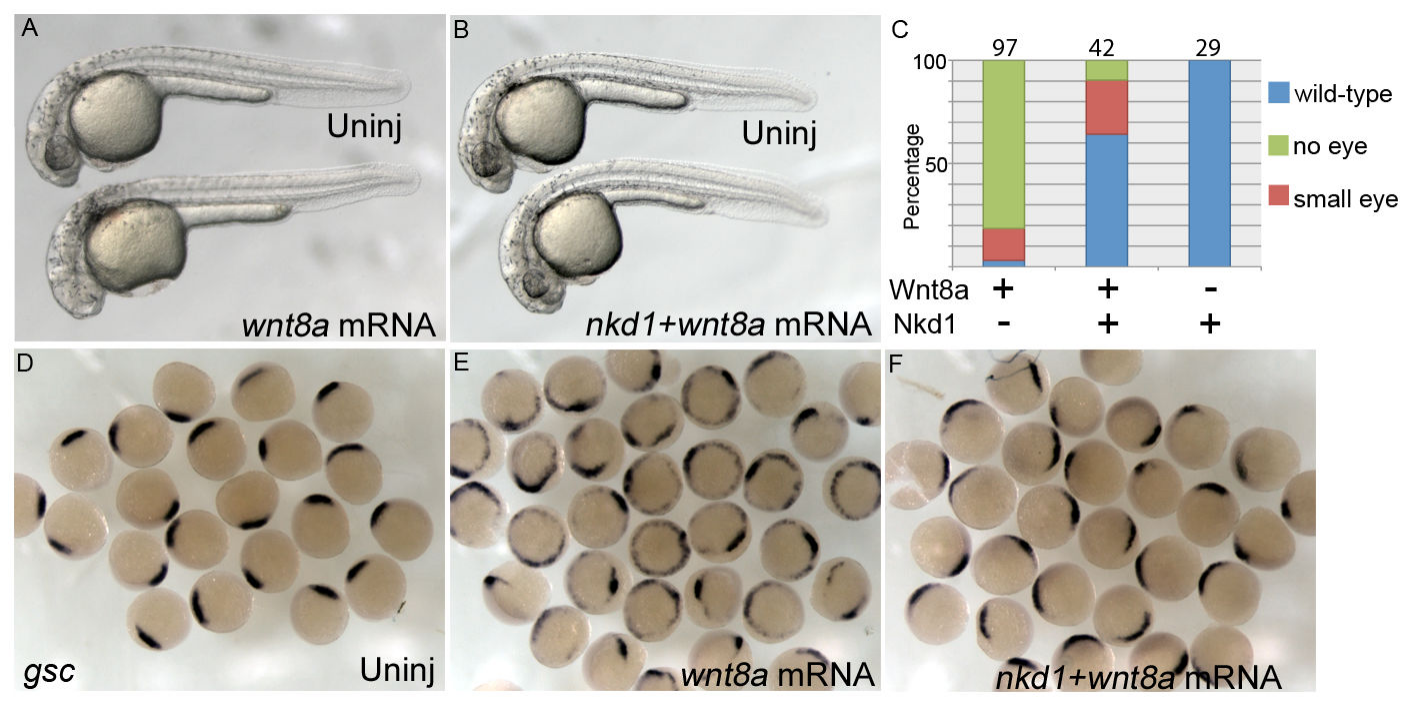
Nkd1 is sufficient to antagonize ectopic Wnt8a. Overexpression of Wnt8a (25pg) results in an eyeless phenotype that can be rescued by co-injection of *nkd1* (A, B) which is quantified in (C). Numbers above each column represent n values. Overexpression of high Wnt8a (200pg) results in ectopic *gsc* expression along the ventral-lateral domain at 50% epiboly (E). Co-injection of high *wnt8a* with *nkd1* mRNAs dramatically reduces the ectopic *gsc* expression, but leaves the putative endogenous *gsc* domain intact (F).

While Nkd1 has little apparent affect on overall development, it is still sufficient to antagonize Wnt signaling, although to varying degrees dependent on if it is endogenous or exogenous Wnt signaling (Figure 2). Overexpression of Wnt8 in zebrafish embryos results in an eyeless phenotype at 1 dpf, which is effectively rescued by Nkd1 and overall development appears normal, albeit with a smaller eye (Figure 2A–C). Similarly, Wnt8 can induce ectopic *gsc* expression at 50% epiboly, which is also effectively reduced by the addition of Nkd1 (Figure 2D–F). Note however, that there is also a subtle effect on endogenous Wnt signaling: expansion of dorsal *gsc* expression (Figure 2F). This is consistent with our previous observations, where Nkd1 overexpression can antagonize endgonous Wnt8 signaling along the ventro-lateral domain at 50% epiboly, resulting in expanded expression of *gsc*, a dorsal organizer marker [12] (Figure 2F). This expansion must be corrected for later in development since we do not observe a dorsalized phenotype at 1 dpf (Figure 2B). This dichotomy in the effect of exogenous Nkd1 on endogenous or ectopic Wnt signaling suggests that the main effect of exogenous Nkd1 is to inhibit ectopic Wnt signaling.

**Figure 3:**
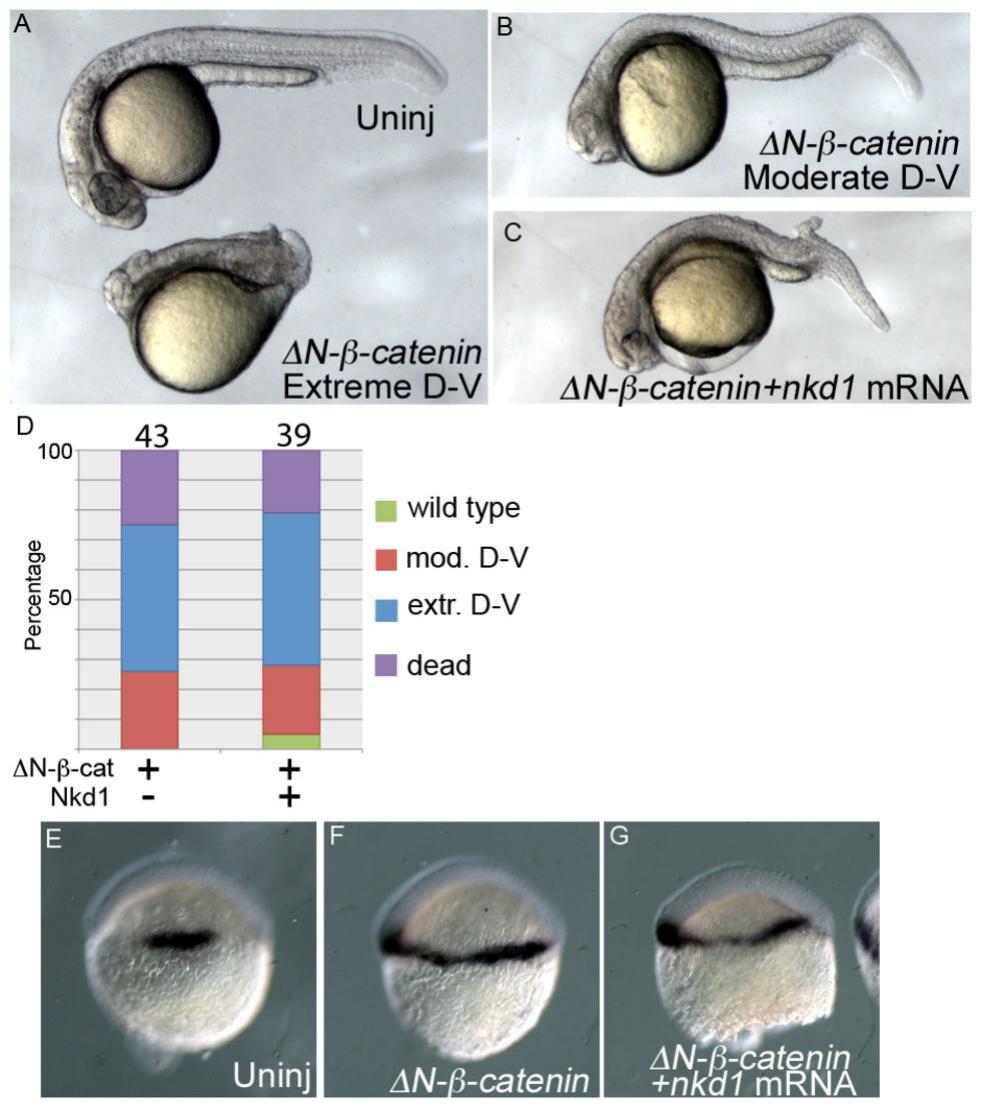
Nkd1 is insufficient to antagonize constitutively active β-catenin. Overexpression of ∆N-β-catenin results in a spectrum of dorsal-ventral (D–V) phenotypes ranging from a severe phenotype (A), which shows dramatic reduction in both dorsal and ventral structures to a moderate phenotype (B), which have reduced dorsal and ventral structures. The addition of Nkd1 does not ameliorate the effect of ∆N-β-catenin. The distribution of phenotypes in *∆N-β-catenin* and *nkd1* injections is quantified in (D), with numbers above each column representing n values. Consistent with the 1 dpf phenotype, ∆N-β-catenin overexpression results in expansion of *gsc* expression (E, F), which is not reduced by the addition of Nkd1 (G) (uninj n=40; *∆N-*β-*catenin* n=32; *∆N-*β-*catenin+nkd1* n=34).

Nkd1 interacts with both Dvl and β-catenin and appears to function by inhibiting the nuclear accumulation of β-catenin [13]. However, we found that Nkd1 is not sufficient to antagonize constitutively active β-catenin. Overexpression of stable β-catenin (∆N-β-catenin) results in severe dorsal-ventral patterning defects (Figure 3A, B, D) and co-expression of Nkd1 with ∆N-β-catenin does not significantly alter this. In agreement, ectopic *gsc* induced by ∆N-β-catenin is maintained even in the presence of excess Nkd1 (Figure 3E–G; Exp shown, E: Uninj n=26; F: ∆N-β-catenin n=32; G: ∆N-β-catenin+Nkd n=34). It is unknown if this N-terminal region of β-catenin is required for interaction with Nkd1.

**Figure 4:**
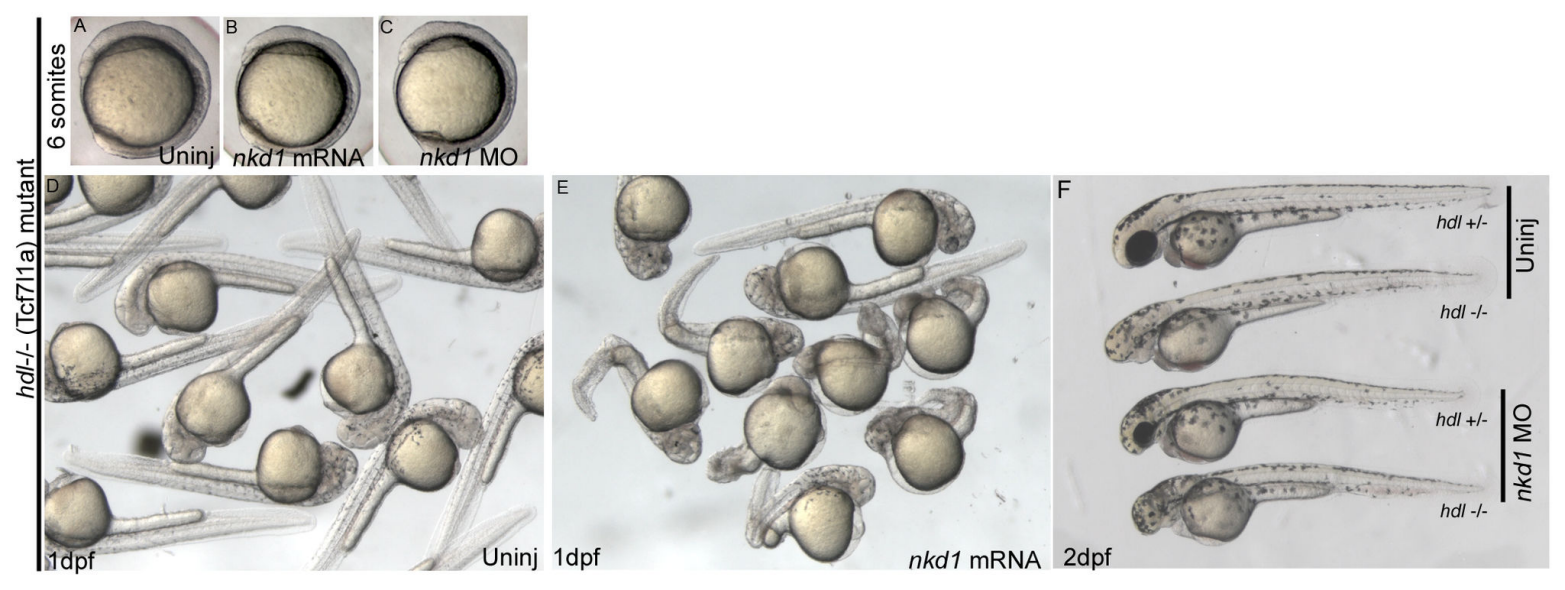
Nkd1 does not rescue the *hdl*/*tcf7l1a* mutant. Homozygous deletion of *tcf7l1a* results in an eyeless phenotype due to activated canonical Wnt signaling (D). Overexpression of Nkd1 in embryos from a homozygous hdl-/- parental cross (B, E) does not rescue the eyeless phenotype (E) and does not affect development of the early embryo (B), although at 1 dpf, *nkd1* injected embryos typically have a kinked axis. Injection of *nkd1* MO into embryos from a *hdl* +/- X *hdl* -/- parental cross has no affect on early (C) or 1 dpf (F) development (Uninj *hdl* +/- n= 14, *hdl* -/-n= 16; Nkd1 MO injected *hdl* +/- n= 15, *hdl* -/-n=22). Experiments in embryos from homozygous hdl-/- parental crosses had similar results (not shown).

To further explore the function of Nkd1, we tested whether Nkd1 was sufficient to inhibit ectopic Wnt signaling in the headless (*hdl/tcf7l1a* (*tcf3*)) mutant, which has a mutation in the *tcf7l1a* transcription factor gene. In the absence of β-catenin, *tcf7l1a* is a transcriptional repressor and only in the presence of activated Wnt signaling and stabilized β-catenin is the inhibition released and transcription initiated. However, a frameshift mutation in the *hdl* mutant results in a truncated protein that can still bind β-catenin but cannot bind DNA [33]. As such the *hdl* mutant mimics the Wnt8a overexpression phenotype in that both lack eyes (Figure 2A c/w Figure 4D). Knockdown of Nkd1 or overexpression of Nkd1 has no effect on the overall development of the early *hdl* mutant embryo (Figure 4A–C). At 1 dpf, ectopic Nkd1 results in a kinked axis phenotype, but no rescue of the eyeless phenotype (Figure 4E). Knockdown of Nkd1 has no effect other than slight necrosis at 1 dpf (Figure 4F). This data is consistent with the role of Nkd1 acting between the ligand-receptor complex and the nuclear accumulation of β-catenin [13].

### Wnt/PCP mutants are sensitive to Nkd1

Previously we determined that Nkd1 can inhibit non-canonical Wnt/PCP signaling independent of Nkd1’s role in canonical Wnt/β-catenin signaling [12]. Furthermore, it is well established that the Wnt/PCP pathway can antagonize canonical Wnt/β-catenin signaling [23–27]. Dependent on where in the signaling cascade this antagonism occurs, we would predict that some Wnt/PCP mutants would be more sensitive than others to alterations in canonical Wnt signaling. Coincident with this, homeostatic levels of canonical Wnt signaling may fluctuate more in Wnt/PCP mutants than in wild type embryos, which in turn may uncover a more active role for Nkd1. There are several lines of evidence supporting the sensitivity of Wnt/PCP mutants to canonical Wnt signaling [24,34–36]. For example in Drosophila, the vacuolar (V)-ATPase proton pump (VhaPRR) is required in the Wnt/PCP pathway, but was also found to restrict the expansion of Wg morphogen [34]. In zebrafish, the maternal-zygotic *wnt5a* mutant, *pipetail* (*ppt*), has a highly dorsalized phenotype [24] and loss of *vangl2* (*strabismus* or *tri* mutants) results in embryos highly sensitive to canonical Wnt signaling [35,36]. Importantly, in these zebrafish mutants, activation of, or sensitivity to, canonical Wnt signaling (or dorsalization) occurs before the onset of gastrulation, strongly suggesting that Wnt/PCP specific components regulate canonical Wnt signaling prior to activation of the Wnt/PCP pathway.

**Figure 5:**
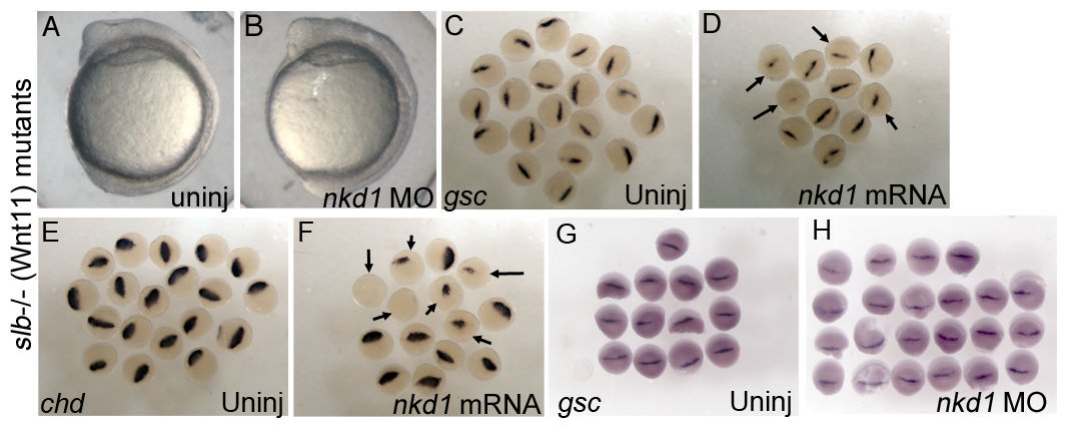
The *silberblick* (*Wnt11*) mutant is sensitive to Nkd1. The *slb* mutant undergoes normal convergence and extension (A), which is not affected by knockdown of Nkd1 with morpholinos (B). Overexpression of Nkd1 in slb-/- reduces *gsc* (D) and *chd* (F) expression (arrows), relative to controls (C, E) at 50% epiboly (Nkd1 injected: 47% of embryos with reduced *chd* expression (n=81); 50% of embryos with reduced *gsc* expression (n=46)). In contrast, knockdown of Nkd1 results in a slight expansion of *gsc* expression at 30% epiboly (G: ave *gsc* width=0.35 mm; n=13, H: ave *gsc* width=0.38 mm; n=22). All embryos are homozygous *slb*, derived from homozygous *slb* parents.

To determine if Nkd1 has a more active role in Wnt/PCP mutants, we overexpressed and knocked down Nkd1 function in several Wnt/PCP mutants. Knockdown of Nkd1 in embryos from wnt11-/- (*silberblick*, *slb*) homozygous parents did not have a dorsalizing effect on somitogenesis stage embryos (Figure 5 A, B). At later stages, the necrotic effect of the *nkd1* MO prevented accurate analysis (not shown). However, by measuring the width of *gsc* expression at 30% epiboly, Nkd1 MO resulted in a slightly expanded organizer (Figure 5G, H), consistent with our previous results in wild type embryos [12].

In contrast, overexpression of Nkd1 resulted in a reduction in expression of both *gsc* and *chd* genes at 50% epiboly, more so than observed in wild type embryos (Figure 5 C–F) [12]. As we have previously shown, overexpression of Nkd1 in *slb* mutants resulted in increased cyclopia at 1 dpf [12] (and not shown), consistent with its role in Wnt/PCP signaling. Thus, the reduced *gsc* and *chd* expression in these mutants in the presence of excess Nkd1 suggest it may have a more active role in regulating canonical Wnt signaling in the dorsal organizer in this Wnt/PCP mutant compared to wild type embryos. This is because in wild type embryos we mainly observe decreased *gsc*/*chd* expression only when Nkd1 is injected early, prior to fertilization, affecting maternal Wnt/β-catenin signaling. In more standard 1-2 cell stage injections, overexpression of Nkd1 results in expanded *gsc*/*chd* expression due to its ability to antagonize zygotic, ventral-lateral Wnt8 signaling [12]. Interestingly, even though Nkd1 was sufficient to reduce the expression of *gsc*/*chd* in *slb* mutants, both markers of the dorsal organizer, we did not observe ventralized embryos at 1 dpf. This is likely due to the short half-life of Nkd1, which would allow *gsc* and *chd* expression to recover later in development [37] (data not shown).

We also tested the sensitivity of this mutant to canonical Wnt signaling. Overexpression of Wnt8a resulted in a slight increase in organizer gene expression but had other curious effects at 1 dpf. In multiple experiments, 50% of the embryos died by 1 dpf and of the 50% that remained, 37% had no eye whereas wild type embryo controls had 75% with no eye (*slb* n=69; wt n=92). The surviving embryos also had varying degrees of dorsalization and slight rescue of cyclopia (not shown). Taken together, this suggests that *slb* mutant embryos are more sensitive to canonical Wnt signaling and that this sensitivity increases the antagonistic activity of Nkd1. Furthermore, Wnt8 may also affect the Wnt/PCP pathway, which is currently being investigated.

**Figure 6:**
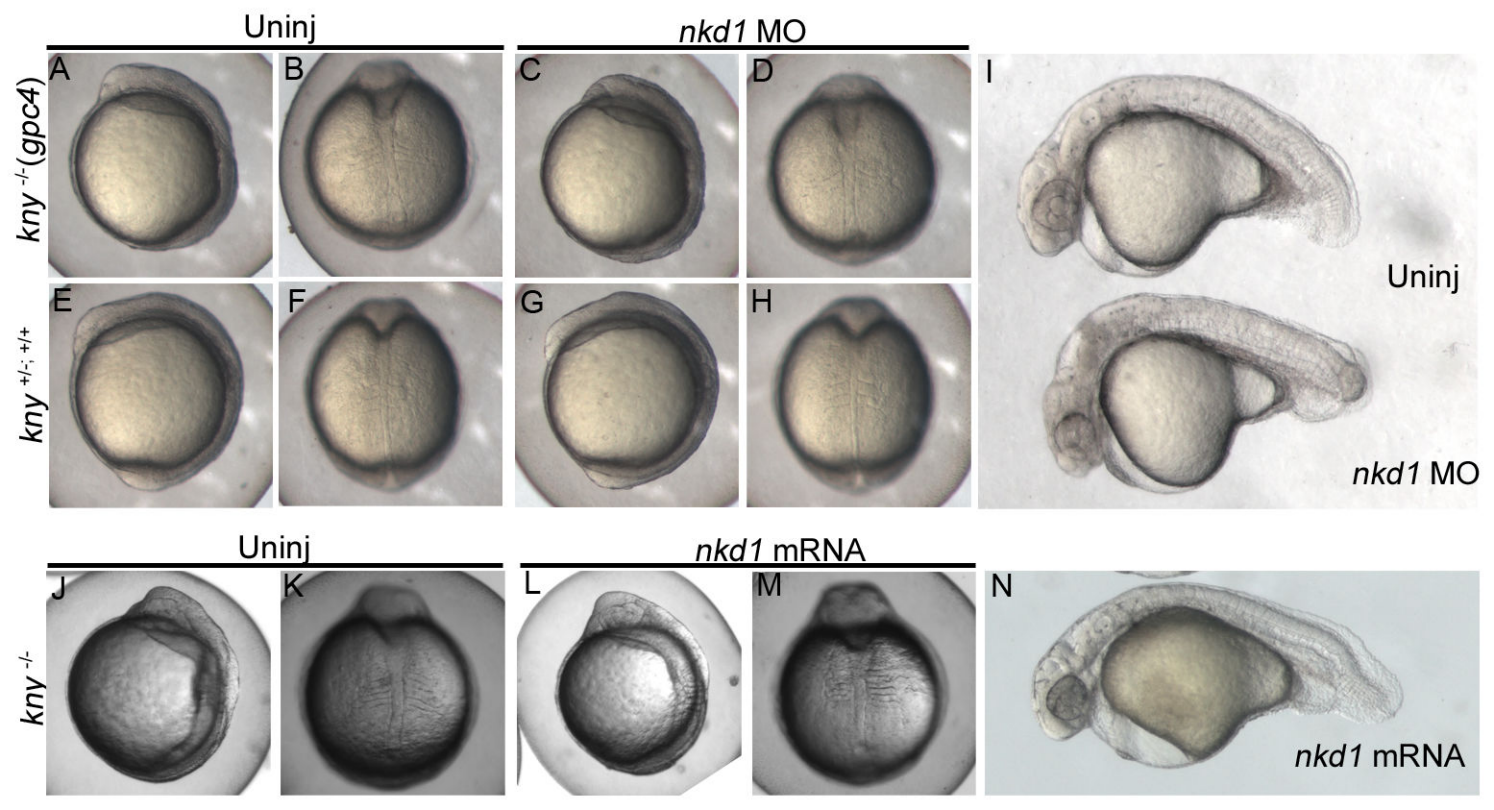
The *knypek* (glypican 6) mutant is not sensitive to Nkd1. Knockdown of Nkd1 in *kny* -/- (C, D) or in *kny* +/+; +/- (G, H) does not have any affect on the *kny* mutant phenotype during somitogenesis (A–H) or at 1 dpf (I; n=19 kny-/-). Note the high levels of neural necrosis in *nkd1* MO injected embryos at 1 dpf (I). Consistent with the lack of sensitivity, overexpression of Nkd1 has no obvious effects during early somitogenesis (J–M) or at 1 dpf (N; n=31 *kny* -/-). There is no change in the ratio of wild-type: mutant phenotypes.

We next tested the glypican mutant, *knypek/glypican 4* (*kny/gpc4*). Overexpression or knockdown of Nkd1 did not have any effect on the development or morphology of somitogenesis stage or 1 dpf embryos (aside from MO toxicity) (Figure 6). In addition, overexpression of *wnt8a* resulted in an eyeless phenotype similar to injections into wild type embryos but otherwise Wnt8a had no effect on the *kny* phenotype and thus we concluded that the *kny* mutant is not sensitive to canonical Wnt signaling. Due to this lack of phenotype we did not determine if Nkd1 or Wnt8a affected organizer gene expression.

**Figure 7:**
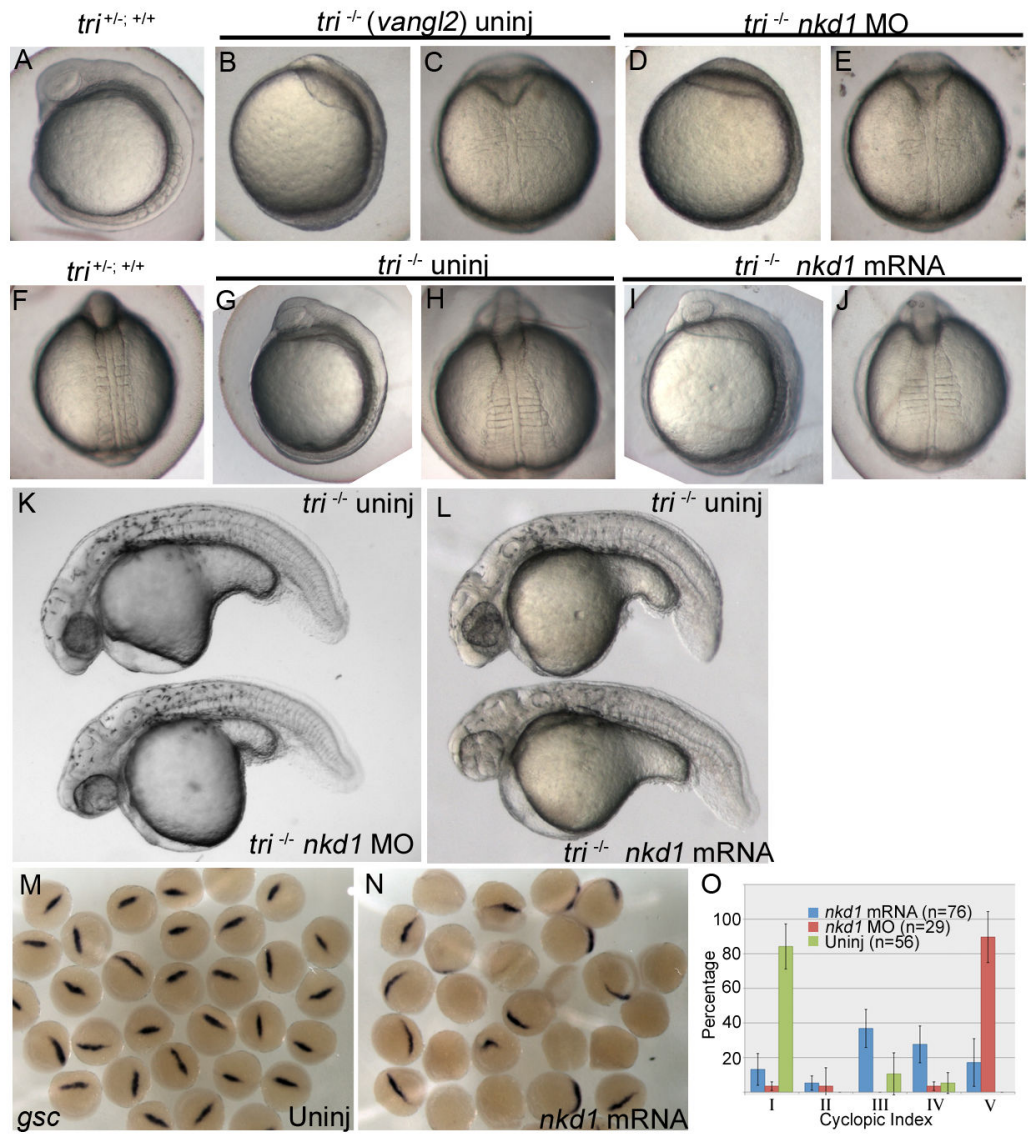
*Trilobite* (*vangl2*) mutants are sensitive to Nkd1. Knockdown of Nkd1 with morpholinos (D, E) or overexpression of Nkd1 (I, J) does not have an obvious effect during early somitogenesis. However, at 1 dpf, knockdown or overexpression of Nkd1 in *tri* mutants results in an increase in cyclopia (K, L, O). Before the onset of gastrulation, at 50% epiboly, embryos generated from a tri+/- X tri+/- parental cross are sensitive to Nkd1 overexpression, demonstrated by a reduction or absence of *gsc* expression (M; n=32, N; 56% of embryos with reduced expression, n=25). (O) The cyclopic index was calculated using criteria established in Marlow et al., 1998 [43]. n values reflect the number of tri-/- embryos. Error bars represent standard error.

Lastly we determined the effect of Nkd1 in the *trilobite/vang-like 2 tri/vangl2*) (also known as *strabismus/stbm*) mutant, which has a mutation in the transmembrane *vangl2* gene [36,38]. Vangl2 is specific for Wnt/PCP and is involved in cell polarity and directed cell migration [38–41]. Knockdown of Nkd1 or overexpression of Nkd1 does not appear to affect development of the early somitogenesis stage embryo (Figure 7 A–J). However, overexpression and knockdown of Nkd1 results in increased cyclopia (Figure 7 K,L,O). Cyclopia results from decreased extension of the axial mesoderm and is a classical Wnt/PCP phenotype (eg. Slb/Wnt11) [42,43]. It is also well established that inhibiting or overactivation of Wnt/PCP gives similar phenotypes, so observing similar effects with knockdown and overexpression of Nkd1 in *tri* mutants is consistent with Nkd1 affecting Wnt/PCP signaling [42,44,45]. Thus, similar to *slb* mutants, Nkd1 affects the expressivity of the Wnt/PCP mutant phenotypes, but in the case of *kny*, Nkd1 has no effect at all.

Because Vangl2 has been implicated in regulating canonical Wnt signaling we looked at the ability of ectopic Nkd1 to reduce the expression of dorsal organizer markers. Consistent with our results using the *slb* mutant, we observed a significant decrease in *gsc* gene expression in more than 50% of the embryos obtained from *tri* heterozygote crosses injected with *nkd1* mRNA (Figure 7 M, N). Considering we crossed heterozygote *tri* parents, we expected 25% of the embryos (tri-/-) to have a phenotype, the remaining 75% (*tri* +/-; +/+) to be similar to *nkd1* injections into wild type embryos and have slightly expanded *gsc* expression [12]. These results suggest that heterozygous *tri* embryos are haploinsufficient for the *tri* gene with respect to its ability to regulate canonical Wnt signaling, but the genotype of the individual embryos has not been determined. The effect of Nkd1 on dorsal gene expression in *tri* mutants is similar to, but greater than, that observed in *slb* mutants from homozygous mutant parental crosses. We conclude that Nkd1 function in canonical Wnt signaling is more active in *vangl2* mutants compared to *slb* mutants and wild type embryos, potentially as a result of reduced Wnt/PCP control over canonical Wnt/β-catenin signaling. Curiously, and as mentioned above for the *slb* mutants, the majority of the embryos with reduced or absent *gsc* expression must recover later in development, because we did not observe significant D–V patterning defects at 1 dpf (Figure 7L).

**Figure 8:**
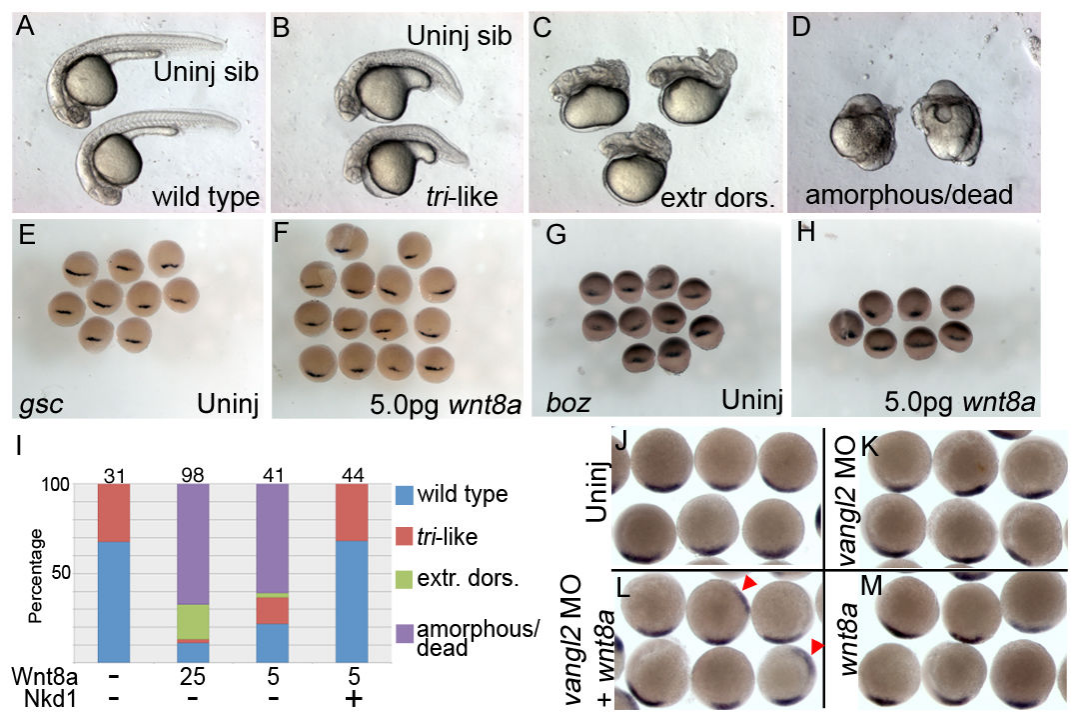
Trilobite (*vangl2*) mutants are highly sensitive to canonical signaling. Injection of a low dose (5pg) of *wnt8a* into embryos from a tri+/- X tri+/- parental cross results in extremely dorsalized embryos and significant lethality (A–D, I). Different classes of phenotypes are shown in (A–D) with an uninjected wild-type sibling shown at the top in (A), and an uninjected tri mutant shown at the top in (B) for comparison. Addition of Nkd1 is capable of fully suppressing the *wnt8a* overexpression lethality and dorsalization (I). n values are for all genotypes. In contrast to the extreme phenotypes seen at 1 dpf, there is no effect of Wnt8a on the early organizer (E–H) shown by expression of *gsc* (E, F) and *bozozok* (*boz*) (G, H) an early and direct transcription target of maternal Wnt signaling. (J–M) Injection of *vangl2* MO does not alter the expression of *gsc* (K), nor does the low level of *wnt8a* (M). However, co-injection of *vangl2* MO and *wnt8a* results in ectopic *gsc* expression about 2-5% of the time (red arrowheads, n= 21).

To explore the function of Nkd1 and the sensitivity of *vangl2* mutants further, we overexpressed the canonical Wnt ligand Wnt8a. A dose of *wnt8a* that causes an eyeless phenotype in 75% of wild type embryos, but no dorsalization (Figure 2 A,C) was lethal to the majority of embryos from a tri+/- heterozygote cross (Figure 8D, I). Even a lower dose of *wnt8a* that has no affect in wild type embryos caused significant lethality in more than 75% of the embryos (Figure 8D, I). This result was repeated in two different *tri* alleles with nearly identical results (*m209* and *m747*, not shown). Consistent with the effect of Nkd1 on *gsc* expression in these embryos, this suggests that a reduction in the maternal contribution of Vangl2 is sufficient to sensitize these embryos to Wnt signaling. At the lower dose of *wnt8a*, approximately 15% of the embryos had a strong dorsalization phenotype which phenocopies the maternal *wnt5a* mutant, *ppt* (Figure 8C, I) [24]. Therefore, we hypothesized that there would be a significant increase in dorsal gene expression in *wnt8a* overexpressing embryos, but the levels of *gsc* and *boz*, a direct target of maternal Wnt signaling [18], where relatively normal at 40% epiboly (Figure 8E–H).

To further explore the sensitivity of *tri* to canonical Wnt signaling, we used antisense oligonucleotide morpholino’s to knockdown Vangl2 protein levels, which effectively recapitulates the *tri* phenotype [35,38] and data not shown. Co-injection of *vangl2* MO with *wnt8a* results in significant lethality by 1 dpf (not shown), similar to the effect of Wnt8a in *tri* mutants. We also determined the effect on dorsal-ventral patterning by assaying the expression of *gsc*. Injection of *vangl2* MO or *wnt8a* individually did not have a significant affect on the size of the *gsc* expression domain (Figure 8J, K, M), which is not surprising considering we did not observe changes in *gsc* or *boz* expression in the *tri* mutants. However, co-injection of *vangl2* MO with *wnt8a* would occasionally result in ectopic *gsc* (2-5%), but the endogenous domain was not significantly perturbed (Figure 8L). This inconsistency between highly dorsalized 1 dpf embryos and a lack of increase in dorsal gene expression, while surprising, is consistent with previous reports. Knockdown of Rack1, which is required for Vangl2 membrane localization and inhibition of canonical Wnt signaling, also had low percentages of ectopic chordin (dorsal genetic marker) and ectopic axis [35]. There are at least two possible explanations for this inconsistency. First, is the possibility that overexpression of Wnt8a is insufficient to expand *boz* expression based simply on the timing of transcription and translation of the *wnt8a* gene to accumulate to sufficient levels. Second, *gsc*, and to a lesser extent, *chd*, are under transcriptional control of the Nodal pathway as well as the Wnt pathway and thus may not be the best readout of activated Wnt signaling [30]. As a more direct readout of Wnt activity, Park and Moon [36] used a TCF-luciferase reporter and found that the *vangl2* mutant (*stbm*) was extremely sensitive to canonical signaling having significantly higher levels of luciferase in the presence of Wnt8a. In agreement with our observations, they found that by itself the *vangl2* mutant does not exhibit increased Wnt signaling [36]. We are currently exploring the expression of other Wnt targets in this system. Nevertheless, the presence of ectopic *gsc* (Figure 8L), *chd* [35] and increased canonical Wnt reporter expression [36] argues that canonical Wnt signaling is influenced by Vangl2 or other Wnt/PCP proteins such as Wnt5a. Reduced Wnt/PCP proteins (Vangl2, Wnt11 and Wnt5a in particular) makes the embryo highly susceptible to increased canonical Wnt signaling.

To test the function of Nkd1 in this excessive Wnt signaling scenario, we co-injected low *wnt8a* and *nkd1* mRNAs into *tri* embryos from a heterozygote cross. We found that Nkd1 completely rescued the lethality and dorsalization phenotypes induced by Wnt8a (Figure 8I) and these embryos looked either completely wild-type or tri-like in the correct mendelian ratios (Figure 8A, B, I).

## Discussion

Here we describe the effect of Nkd1 in different genetic backgrounds. We have shown that knockdown or overexpression of Nkd1 does not have an overt phenotype in wild type embryos at 1 day post fertilization. At first, this seems at odds with a Wnt antagonist, but it is consistent with phenotypes seen in both mice and flies. The single *nkd1* or double *nkd1*/2 knockout mice do not have any gross phenotype, except for a mild craniofacial morphology defects [22]. In Drosophila, knockout of *nkd* results in increased Wnt activity during larval stages and the formation of naked cuticles, but curiously, Nkd does not have other gross defects in known Wnt expressing domains [21].

Given the passive nature of Nkd1 in wild type embryos and in some mutants, our results in *slb* and *tri* mutants were surprising. It strongly suggests that Nkd1 functions as a passive antagonist, and only when the levels of Wnt signaling exceed a certain threshold does Nkd1 become active. This could explain why the *nkd1*/2 knockout mouse has no apparent phenotype outside of cranial-facial dysmorphology [22]. It is possible that these mice are not challenged sufficiently to observe a phentoype. In support of this, compared to other Wnt antagonists, Nkd1 does not dramatically alter the endogenous expression domains of Wnt signaling [28–30]. The nature of this is unclear, but suggests that the endogenous Wnt signaling domain is refractory to, or protected from, Nkd1 activity. The Wnt signalosome model, which involves the sustained clustering of Wnt receptors [46–48], may prevent Nkd1 from gaining access to the membrane simply by stoichiometry. It follows then that ectopic expression of *wnt8a* may not maintain a sustained signalosome, allowing Nkd1 to attenuate this transient signal. Importantly, we have previously determined that membrane localization of Nkd1 is required for its function [13,37]. While the function of Nkd1 at the membrane is unclear, it is possible that in the *vangl2* mutants, the reduction in total membrane protein may allow Nkd1 access to important signaling events. Unfortunately, how Vangl2 and other Wnt/PCP mutants antagonize canonical Wnt signaling is unclear and may occur at the level of the membrane bound receptors or in the cytoplasm [23–27]. Future work will take advantage of the Wnt/PCP mutant sensitivity to canonical Wnt signaling to determine exactly how Nkd1 functions to antagonize Wnt signaling and how the Wnt/PCP pathway restricts/reduces or inhibits Wnt/β-catenin signaling. In conclusion, our results support a model whereby Nkd1 acts as a passive Wnt antagonist. Under homeostatic levels of Wnt signalling, Nkd1 is passive, not affecting endogenous Wnt signalling. Only when Wnt signaling breaches a certain threshold or becomes unstable in some Wnt/PCP mutants does Nkd1 become an active and potent inhibitor of Wnt signalling.

## Methods

### Ethics Statement

All animal work has been done in accordance with national and international guidelines and has been approved by the University of Guelph Animal Care Committee AUP #1295.

### Fish maintenance

Adult zebrafish and embryos were raised at 28.5^°^C and were staged by anatomical criteria or hours or days post fertilization (hpf and dpf, respectively) according to Kimmel et al [49]. Maternal zygotic or zygotic *hdl*, *slb* ^tz215^, *kny* ^m119^, *tri* ^m207^ or *tri* ^m747^, *kny* ^m119^ homozygous or heterozygous embryos (all in AB* backgrounds) were collected from pairwise matings of homozygous or heterozygous adults, respectively.

### Whole-mount in situ hybridization

Staged embryos were fixed overnight at 4^°^C in phosphate-buffered saline solution containing 4% paraformaldehyde. In situ hybridization was carried out as described by Thisse et al. (1999). The following antisense digoxigenin-labeled probes were used: *chordin* [50], *goosecoid* [51,52] and *bozozok* [18]. Probes were synthesized using T7, T3, or SP6 RNA polymerases (Ambion) and precipitated with LiCl and EtOH and resuspended in RNase free . by spectrophotometry and quality was assayed using gel electrophoresis.

Width of gsc expression was measured by ImageJ

### mRNA and morpholino oligonucleotide injections

Capped mRNA was synthesized using Ambion’s mMessage mMachine kit. Following transcription, the RNA was purified over a G-50 sephadex column (Roche) and diluted in RNase free water, and its quantity and quality was analyzed as described above. The following mRNA concentrations were used: *wnt8a* [32,53]: Figure 2E, 200 pg; Figure 2A–C, 25 pg; Figure 8I, L, M, 25 pg; 25 pg for *kny* and *slb* mutant injections (not shown); Figure 8A–I, 5 pg; ∆N-β-catenin 10pg; *nkd1* mRNA: 800 pg, except for Figure 3 G: 1600pg; *nkd1* ATG MO 4 ng (5’-GAAGTTTACCCATTTCTCTGCATCG-3’); Vangl2 MO has been previously described [36]. 4 ng of Vangl2 MO was used. MO’s and mRNA’s were pressure injected into the yolk cell of one- to two-cell-stage embryos. Antisense morpholino oligonucleotides (MOs) were dissolved and diluted in water containing 1% phenol red. At all but the 0.5 ng dose tested for the Nkd1 MO, there was non-specific necrosis by mid-segmentation stages. All experiments were repeated at least once.
